# Computer-vision based analysis of the neurosurgical scene – A systematic review

**DOI:** 10.1016/j.bas.2023.102706

**Published:** 2023-11-07

**Authors:** Félix Buyck, Jef Vandemeulebroucke, Jakub Ceranka, Frederick Van Gestel, Jan Frederick Cornelius, Johnny Duerinck, Michaël Bruneau

**Affiliations:** a Department of Neurosurgery, Universitair Ziekenhuis Brussel (UZ Brussel), 1090, Brussels, Belgium; b Vrije Universiteit Brussel (VUB), Research group Center For Neurosciences (C4N-NEUR), 1090, Brussels, Belgium; c Vrije Universiteit Brussel (VUB), Department of Electronics and Informatics (ETRO), 1050, Brussels, Belgium; d Department of Radiology, Universitair Ziekenhuis Brussel (UZ Brussel), 1090, Brussels, Belgium; e imec, 3001, Leuven, Belgium; f Department of Neurosurgery, Medical Faculty, Heinrich-Heine-University, 40225, Düsseldorf, Germany

**Keywords:** computer vision, Surgical videos, Automated detection, Surgical instruments, Surgical phase recognition, Neuroanatomy

## Abstract

**Introduction:**

With increasing use of robotic surgical adjuncts, artificial intelligence and augmented reality in neurosurgery, the automated analysis of digital images and videos acquired over various procedures becomes a subject of increased interest. While several computer vision (CV) methods have been developed and implemented for analyzing surgical scenes, few studies have been dedicated to neurosurgery.

**Research question:**

In this work, we present a systematic literature review focusing on CV methodologies specifically applied to the analysis of neurosurgical procedures based on intra-operative images and videos. Additionally, we provide recommendations for the future developments of CV models in neurosurgery.

**Material and methods:**

We conducted a systematic literature search in multiple databases until January 17, 2023, including Web of Science, PubMed, IEEE Xplore, Embase, and SpringerLink.

**Results:**

We identified 17 studies employing CV algorithms on neurosurgical videos/images. The most common applications of CV were tool and neuroanatomical structure detection or characterization, and to a lesser extent, surgical workflow analysis. Convolutional neural networks (CNN) were the most frequently utilized architecture for CV models (65%), demonstrating superior performances in tool detection and segmentation. In particular, mask recurrent-CNN manifested most robust performance outcomes across different modalities.

**Discussion and conclusion:**

Our systematic review demonstrates that CV models have been reported that can effectively detect and differentiate tools, surgical phases, neuroanatomical structures, as well as critical events in complex neurosurgical scenes with accuracies above 95%. Automated tool recognition contributes to objective characterization and assessment of surgical performance, with potential applications in neurosurgical training and intra-operative safety management.

## Highlights

• Robotic surgery & AI in neurosurgery raise interest in automated surgical image analysis.
• Systematic review: Computer vision methods for automated analysis of digital images and videos in neurosurgery.
• Studies report the use of CV models for the analysis of tools, neuroanatomy, workflow and critical events in neurosurgery.
• CNN predominant (65%) for accurate tool detection & segmentation.
• CV models may aid in objective surgical assessment & training enhancement.

## 1 Introduction

### 1.1 Background

The digital revolution is a well-known phenomenon that emanated from the introduction of computers in healthcare in the late 80's (DeTore, 1988; Giudice and Famà, 2020). At present, zettabyte is the scale on which healthcare data is expressed (Raju et al., 2020). In 2020, the total capacity of medical data was estimated at 2.314 zettabytes, equivalent of 2,314,000,000,000 gigabytes (Alsuliman et al., 2020a). At present, we have come to enter the era of big healthcare data. Despite the numerous prospects such data endows, there are also considerable hurdles associated with it's processing (Panesar et al., 2020; Davenport and Kalakota, 2019). To this end, machine learning (ML), a subfield of artificial intelligence, and more recently, deep learning (DL), a specific type of ML, have gradually found their way into the healthcare system. These methods have the potential to improve diagnostic and prognostic operations, facilitate clinical decision-making, and improve the operative workflow (partial automation, intelligent robots, etc) (Dagi et al., 2021).

Since the improvements of ML and DL-based data processing techniques have made analysing the exponentially growing volume of medical data possible, this has sparked an increased interest in analysis of medical images such as magnetic resonance, computed tomography and ultrasound imaging but also of digital images and videos acquired from the surgical scene.

### 1.2 Computer vision in the operating room

Despite the numerous applications in non-interventional tasks (Raju et al., 2020; Senders et al., 2018a, 2018b, 2018c; Danilov et al., 2020a, 2020b), the clinical implementation of ML in surgical care remains sparse, particularly in neurosurgery. Notwithstanding, there are numerous opportunities for ML within this sector of healthcare. One possible application includes the analysis of the surgical scene for the purpose of quality assessment. Variance in the provided medical care was shown to be a substantial source of errors and complications and may entail a significant physical, mental and economic burden to the patient (Stopa et al., 2019; Meyer et al., 2022; Rolston and Bernstein, 2015; Rolston et al., 2014; Dewan et al., 2018). In this context, it is estimated that approximately 20% of medical errors in neurosurgery occur during an intervention, of which 18–28% emanate from technical or procedural errors (Meyer et al., 2022; Rolston and Bernstein, 2015; Rolston et al., 2014). Surgical videos have proven to be a valuable resource in procedural complication management and neurosurgical training (Sarkiss et al., 2016; Knopf et al., 2020). However, due to the unstructured nature of the data and lack of time and resources, qualitative and quantitative analysis proves cumbersome, ultimately leaving a large amount of this valuable data unused. Computer vision (CV), also known as machine vision, was gradually introduced with the aim of enabling automated surgical assessment.

CV is the computer science which focuses on the use of algorithms that enable computers to analyse and understand graphic data by deriving meaningful information from digital images, videos and other visual inputs20–22. This offers different ways to identify and classify visual features, that may in turn serve as an objective and reproducible frame of reference for surgical performance evaluation and even in neurosurgical training. The comprehension of visual data is established through the use of image processing and pattern recognition (Wiley and Lucas, 2018). Depending on the sensor used, digital images are constituted by pixels which represent the color intensity (red/green/blue pixel values), grayscale intensity, infra-red reflectivity intensity, depth estimation, etc. While these pixels may not carry much meaning individually, structures in an image take shape by looking at groups of pixels and can be detected by considering the patterns and relationships that neighboring pixels form with one another. Traditional CV techniques apply image processing algorithms on the matrix of pixel values to extract features such as edges, corners and textures that allow computer systems to recognize objects within a digital image (What Is Computer Vision, What is Computer Vision). These handcrafted feature extraction techniques are nowadays successfully replaced by convolutional neural networks (CNN) approaches, a type of deep learning networks that works through a hierarchy of interconnected neural network layers. These networks show state-of-the-art performance in CV, have a strong ability to extract complex features that express the image in more detail, and importantly, allow for learning the set of features directly from training data.

In this paper we will not go into detail on the topic of the different learning techniques nor the specifics of various machine or deep learning models that can be applied. Instead, this paper addresses the current status, prospects and challenges in the development of CV models for video analysis in the neurosurgical field and feasibility of CV-assisted surgical performance evaluation.

### 1.3 Fundaments of computer vision

**Fig. 1:**
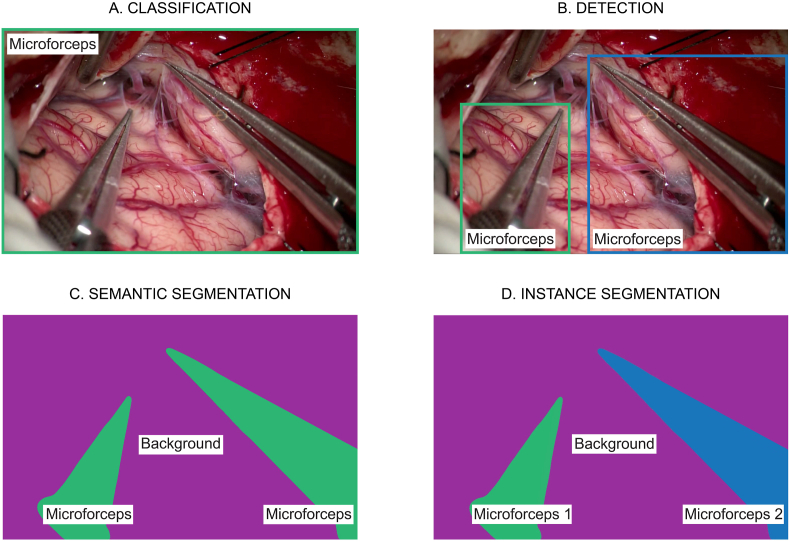
Fundamental outcomes of computer vision: *A. (top left) illustrates how two micro forceps are recognized through classification of images on a frame-wise level. B. (top right) illustrates the coarse localization of the two micro forceps with bounding boxes through object detection. C. (down left) illustrates the detailed localization and mapping of the two micro forceps (green) by labelling all pixels pertaining to microforceps and background (purple) using semantic segmentation. D. (down right) illustrates the detailed localization of the two micro forceps (green & blue) as separate instances with respect to the background using instance segmentation.* (For interpretation of the references to color in this figure legend, the reader is referred to the Web version of this article.)

To further understand the basic principles of CV and interpretation of results we must first elucidate the 4 fundamental tasks that can be performed with CV-based image analysis. More precisely i) Classification ii) Detection iii) Semantic segmentation and iv) Instance segmentation (Fig. 1).

#### 1.3.1 Classification

Classification involves the attribution of one or multiple labels to an image, for example: “there is a scalpel in this image”. Objects or structures are thus recognized in a categorical fashion at frame-level.

#### 1.3.2 Detection

Contrary to classification, detection involves the attribution of one or multiple labels to a region of interest rather than the complete image. This localization remains rudimentary, given that the output is often a rectangular bounding box encompassing the object which may also include background areas that do not correspond to that structure.

#### 1.3.3 Semantic segmentation

Segmentation is similar to detection in the sense that objects are classified as well as localized. However, in this case labels are attributed on a pixel-level. The resulting delineation of object only includes relevant areas contained within the borders of an object and is therefore more precise than bounding-boxes.

#### 1.3.4 Instance segmentation

It is important to note that semantic segmentation does not discriminate between different objects belonging to the same class. Instance segmentation differs from semantic segmentation in the sense that the process allows for recognition of multiple instances of same class, which are delineated separately.

Essentially, the performance of any CV algorithm can be evaluated using a confusion matrix, in which the number of predicted labels is compared against the ground truth. The table shows the number of true-positive, false-positive, true-negative, and false-negative predictions, from which various system performance metrics can be derived. The situation becomes more complex when considering that some algorithms typically output a number between 0 and 1, and that a user-chosen treshold is applied to assign the final label. Every threshold will lead to a new confusion matrix and therefor a new compromise between false positives and false negatives. Similarly, for algorithms aiming at localization, the performance can be evaluated by considering various cut-offs for the vicinity of the prediction and the ground truth.

To ensure adequate measurement of the model performance, it is crucial to report suitable performance metrics, selected in accordance with the image analysis task. Adhering to the recommended terminology outlined within the report concerning the application of image processing metrics by the international multidisciplinary consortium (Maier-Hein et al., 2022), one can distinguish six groups of performance metrics across the different CV functions:

• Per-class counting metrics: a group of validation metrics capturing the performance of each class individually.
• Multi-class counting metrics: a group of validation metrics capturing the performance of all classes as one performance metric value.
• Multi-threshold metrics: On an operator curve, metrics are calculated as a function of a specific value or condition, characterising the trade-off or relationship between different evaluation metrics at specific thresholds. Rather than being based on a static threshold (e.g. for generating the confusion matrix), multi-threshold-based metrics integrate over a range of thresholds, allowing for an in-depth characterization of the systems performance.
• Localization metrics: permit to quantify the correctness of object detection in an image interpretation results, measuring the correspondence between predicted labels and the ground truth.
• Overlap-based metrics: permit to quantify the extent of overlap between predicted object map and the ground truth in object segmentation.
• Boundary-based metrics: permit to quantify distance intervals between predicted object map and the ground truth in object segmentation.

The recommended assessment metrics per function are summarized in Table 1 as described in the “*metrics reloaded”* framework (Maier-Hein et al., 2022).

**Table 1:** Recommended performance metrics per function.

Classification	Detection	Semantic segmentation	Instance segmentation
*Multi-class counting metrics:* Accuracy BA MCC WCK *Per-class counting metrics:* Sensitivity@PPV PPV@Sensitivity Specificity@Sensitivity Sensitivity@specificity F_β_ score LR+ *Multi-threshold metrics:* AP AUROC	*Localization metrics:* IoU IoR Centroid distance Point inside box/mask/approx *Per-class counting metrics:* Sensitivity@PPV PPV@Sensitivity FPPI@Sensitivity Sensitivity@FPPI F_β_ score *Multi-threshold metrics* AP FROC	*Overlap-based metrics* DSC clDice Fβ score IoU *Boundary-based metrics* ASSD IoU HD MASD NSD	*Overlap-based metrics* DSC clDice Fβ score IoU *Boundary-based metrics* ASSD IoU HD MASD NSD *Per-class counting metrics:* Sensitivity@PPV PPV@Sensitivity FPPI@Sensitivity Sensitivity@FPPI F_β_ score Panoptic quality *Localization metrics* IoU IoR *Multi-threshold metrics* AP FROC

**Abbreviations** | Balanced accuracy (BA); Matthews correlation coefficient (MCC); Weighed cohen's kappa (WCK); Positive likelihood ration (LR+); Average precision (AP); Area under the receive operating characteristic curve (AUROC); Free-response receiver operating characteristic score (FROC); Intersection over union (IoU); Intersection over reference (IoR); Dice similarity coefficient (DSC); Center line dice similarity coefficient (clDice); Average symmetric surface distance (ASSD); Hausdorff distance (HD); Mean absolute surface distance (MASD); Normalized surface distance (NSD); False positives per image (FPPI).

Table 1: Recommended performance metrics per function (adapted from Maier-Hein et al. (Maier-Hein et al., 2022). Note that the symbol "@" in the multi-threshold metrics is used to indicate a specific value or condition for a specific metric, thereby characterizing the trade-off or relationship between different evaluation metrics at specific probability threshold points on the Receiver Operating Characteristic (ROC) curves. For example, the term "Sensitivity@Specificity" means the value of Sensitivity when the Specificity reaches a specific threshold or value (e.g. specificity of 0.9). For a graphical explanation of the abovementioned metrics, please refer to the work of Maier-Hein et al. (Maier-Hein et al., 2022).

As the elementary tasks of classification, detection and segmentation provide a basic understanding of visual data, they also form the basis for higher-level image analysis. In surgery, recognition and localization of objects can serve for motion analysis of surgical instruments. Tool velocity, acceleration and jerk are some common examples of handling metrics that can be derived from the localization of surgical instruments. Other functionalities include tool tip recognition, tool positioning and tool interactions. Similarly, recognition of anatomical structures can provide information on their shape (deformation, movement, interruption, etc). On a higher level, image analysis may also contribute to surgical task analysis by means of phase or step recognition.

This could also allow to assess and appraise the surgical performance, thereby facilitating efficient and deliberate surgical training.

### 1.4 Surgical scene interpretation

The potential of surgical data has already been confirmed by several studies in bariatric surgery, urology and ophthalmology, where CV was applied for the identification of human presence, instruments and critical anatomical structures. Understanding of the surgical scene served as an objective tool for surgical performance assessment, prediction of postoperative outcomes, improvement of the operative workflow (in the operating room as well as the operator) and detection of adverse events (Mascagni et al., 2021; Baghdadi et al., 2019; Rahbar et al., 2020; Ward et al., 2021b, 2021c; Hashimoto et al., 2019; Chadebecq et al., 2020; Shimizu et al., 2021; Padoy, 2019; Bamba et al., 2021a; Morita et al., 2019a, 2019b; Zhang et al., 2020; Gong et al., 2021). Despite ample research in other surgical fields, studies of CV in neurosurgery have been limited, especially with regard to the analysis of operative videos. Possible explanations for this include the reduced availability of surgical videos, the lack of imminently deployable clinical applications and the high complexity of the neurosurgical scene and applied instruments.

Although previous papers (Raju et al., 2020; Panesar et al., 2020; Senders et al., 2018a, 2018b, 2018c; Danilov et al., 2020a, 2020b; Alsuliman et al., 2020b; Layard Horsfall et al., 2021) have covered the different ML modalities in neurosurgery, none have provided an overview of these newly emerging CV models. Whether or not CV may adequately assess and reflect the quality of surgical performance in neurosurgery remains unclear. Moreover, as the field of ML continuously evolves it becomes increasingly difficult to keep up with advancements, understand the significance of results or pitfalls of the applied technologies from a neurosurgeon's perspective.

Thus, the primary aim of this systematic study was to provide an overview of the state-of-the-art methodologies in CV, specifically focused on analysis of neurological images or videos. The secondary aim was a systematic comparison of the different model functions, training methods and performances so as to provide recommendations for the development of CV models for the neurosurgical field. Finally, we explore the feasibility and reliability of CV-based surgical performance assessment specificcally.

## 2 Methods

### 2.1 Search Strategy

We performed a literature search on Web of Science, PubMed, IEE Xplore, Embase and SpringerLink up until March 31, 2023. Inclusion and exclusion criteria were established in accordance to the Preferred Reporting Items for Systematic Reviews and Meta-Analyses (PRISMA) guidelines (Page et al., 2021). The goal of this literature review was to identify studies applying CV algorithms on any type of neurosurgical videos or images, for the purpose of achieving automatic recognition of surgical instruments, workflow, critical events and other possible derived functions. The key elements that were actively sought out included “neurosurgery”, “computer vision”, “instrument detection”, “phase detection”, “critical event detection”, “image analysis” and their respective synonyms. Papers were excluded if they contained no full text, the included data did not relate to neurosurgery, or if their objective was not related to the assessment of the surgical scene. Importantly, studies were omitted if analysis was not carried out automatically or performance metrics were not specified.

Once search results were retrieved from all databases mentioned above, potential eligible papers were screened on their title and abstract and duplicates were removed concurrently. Articles that met all inclusion criteria were subsequently reviewed in full before final inclusion.

### 2.2 Data analysis and Synthesis

The following data was extracted from the included papers: [1] Objective of the reported trial [2] Type of footage [3] Type of surgery [4] Subjects [5] Dataset size and accessibility [6] Tool types [7] Tool characterization features [8] Anatomical structures [9] Phases and steps [10] Model architecture [11] Annotation method [12] Data allocation [13] Hyperparameters [14] Model pre-training [15] Output performance. Additionally, the availability of the model code was sought out for each study.

We established an *ad hoc* 3-point scale to assess the overall quality of the study description with respect to the information given to reproduce the results, regardless of the quality of the model output results, underpinned by the completeness (complete/incomplete/unspecified) of reporting of [1] Data [2] Model architecture [3] Model hyperparameters and [4] Performance metrics.

- **High quality** = all 4 topics fully reported
- **Moderate quality** = 2 or 3 topics fully reported
- **Low quality** = 1 or no topic fully reported, 4 topics incompletely reported, 1 or more topics unspecified

Four essential performance metrics were compared (Sidey-Gibbons and Sidey-Gibbons, 2019; Khan et al., 2021a): Accuracy, Precision, Recall and Intersection over Union.

**Table:** 

**Accuracy**: used for classification and detection models - Definition Measures the ratio of correctly classified/detected frames/objects to all predictions. - Formula $\frac{\text{TP}+\text{TN}}{\text{TP}+\text{TN}+\text{FP}+\text{FN}}$.
**Precision** (= Positive Predictive Value): used for classification and detection models - Definition Measures the probability of a predicted classification/detection corresponding to the right frame/object. - Formula $\frac{\text{TP}}{\text{TP}+\text{FP}}$.
**Recall** (=Sensitivity): used for classification and detection models - Definition Indicates the model's ability to correctly classify/detect all the frames of a phase/object. - Formula $\frac{\text{TP}}{\text{TP}+\text{FN}}$.
**Intersection over Union**: used for detection and segmentation models - Definition Measures the degree of correspondence between the predicted detection region and the ground truth. - Formula $\frac{\left|\text{ground}\;\text{truth}\cap \text{bounding}\;\text{box}\right|}{\left|\text{ground}\;\text{truth}\right|+\left|\text{bounding}\;\text{box}\right|-\left|\text{ground}\;\text{truth}\cup \text{bounding}\;\text{box}\right|}$.
Determining the outcome [TP, FP, TN, FN]: - In case of classification, the outcome of output labels is obtained by applying a specific descision threshold to the model output that determines whether or not a class label is assigned to an image. - In case of detection/segmentation, the outcome of the predicted bounding boxes/regions is determined by means of a specific threshold for intersection over union of the prediction framework with the ground truth framework is used.

The goal of the performance comparison was to examine how well CV models perform in recognizing objects in surgical images/videos at frame/object/pixel-wise level. However, as will be shown, the review of the collected studies revealed considerable heterogeneity in the datasets, model tasks and reported performance metrics used, impairing proper statistical analysis. Therefore, studies were compared in a descriptive manner per task type with respect to one specific performance metric.

## 3 Results

**Fig. 2:**
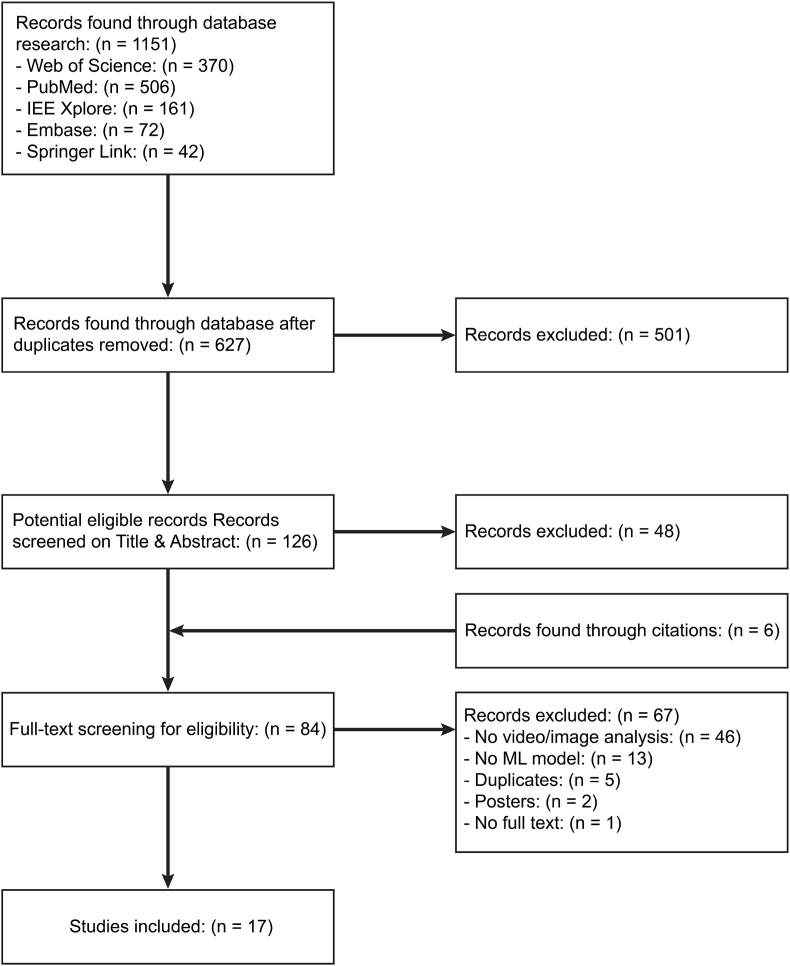
Study selection according to PRISMA guidelines.

**Fig. 3:**
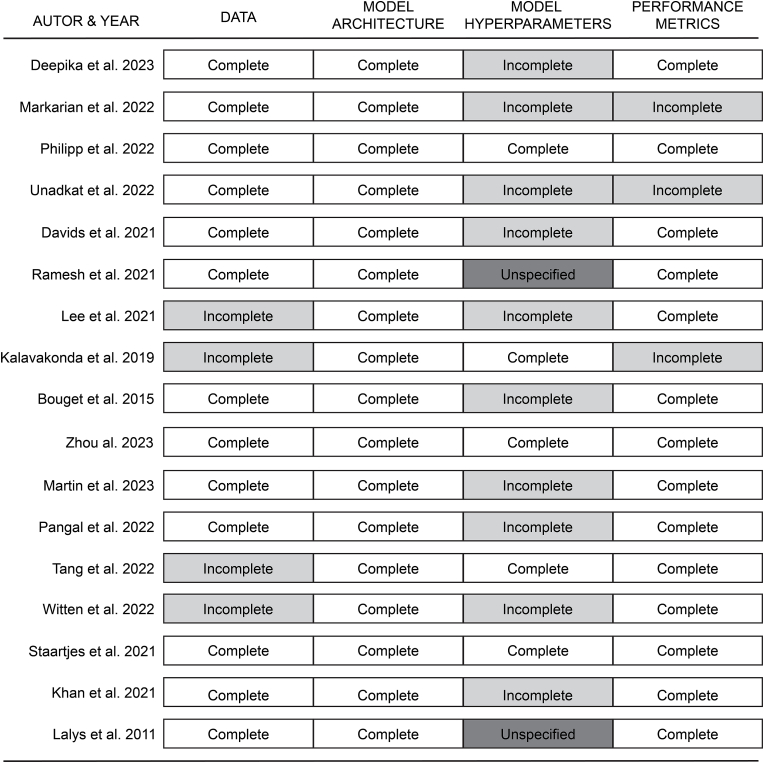
Quality assessment Stacked bar plots displaying the quality of the included studies according to the degree (complete = white; incomplete = light gray; unspecified = dark gray) of reporting data, model architecture, model hyperparameters and performance metrics.

The literature search (Fig. 2) across the different databases yielded a total of 941 results (Web of Science: 370, PubMed: 506, IEE Xplore: 161, Embase: 72, SpringerLink: 42). After discarding duplicate studies from the respective sources, 416 records remained. A total of 126 papers were screened for the title and abstracts of which 84 records were further inspected in terms of their full text for potential eligibility. Through the process of proof-reading, 6 additional eligible studies were identified. In 5 instances, papers were omitted from analysis because studies originated from an identical research project (Pangal et al., 2021a, 2022; Lalys et al., 2010; Philipp et al., 2021; Deepika et al., 2022). In final, 17 studies were included, of which characteristics are summarized in Table 2. Qualitative assessment revealed that the quality of description was “low” in 2 studies, “moderate” in 12 studies and “high” in 3 studies (Fig. 3).

**Table 2:** Baseline characteristics of studies using CV for surgical scene analysis.

Author	Objective	Type of footage	Type of surgery	Subject	Dataset	Tools	Anatomical structures	Phases (Steps)	Dataset access
**Analysis of surgical Instruments**									
Deepika et al., 2023	Recognition & characterization of neurosurgical tools	Microscope	Cranial	Human	7 videos	5	–	–	–
Markarian et al., 2022	Detection of neurosurgical tools	Microscope Endoscope	Cranial Endonasal Cataract Bariatric	Human	39 693 frames	Undefined	–	–	SOCAL NeuroSurgicalTools CaDISv2 M2CAI16-toollocation
Philipp et al., 2022	Recognition of neurosurgical tooltip	Microscope	Cranial Spinal	Human Phantom	16 videos	6	–	–	–
Unadkat et al., 2022	Recognition & characterization of neurosurgical tools	Endoscope	Endonasal	Cadaver	143 videos	4	1	–	SOCAL
Davids et al., 2021a, Davids et al., 2021b	Recognition & characterization of neurosurgical tools Classification of surgical skill level	Microscope	Cranial	Phantom	19 videos	3	–	–	–
Ramesh et al., 2021	Recognition & characterization of neurosurgical tools	Microscope	Cranial	Human	32 videos	6	–	–	–
Lee et al., 2021	Recognition & characterization of neurosurgical tools	Undefined	Undefined	Undefined	950 videos	14	–	–	–
Kalavakonda et al., 2019	Recognition of neurosurgical tools	Undefined	Cranial	Human	5 videos	4	–	–	–
Bouget et al., 2015	Recognition of neurosurgical tools	Microscope	Cranial	Human	14 videos	7	–	–	NeuroSurgicalTools
**Analysis of anatomy and critical events**									
Zhou et al., 2023	Recognition of cerebral aneurysms	Microscope	Cranial	Human	16 videos	–	1	–	MACS
Martin et al., 2023	Quantify retraction of brain tissue	Microscope	Cranial	Human	37 288 frames	3	4	–	–
Pangal et al., 2022	Prediction of blood loss and hemorrhage control success	Endoscope	Endonasal	Cadaver	143 videos	4	1	–	SOCAL
Tang et al., 2022	Recognition of blood loss	Microscope	Cranial	Human Porcine	12 600 frames	–	1	–	–
Witten et al., 2022	Neuroanatomical segmentation	Still images	–	Cadaver	879 images	–	5	–	–
Staartjes et al., 2021	Recognition of anatomical structures	Endoscope	Endonasal	Human	23 videos	–	3	–	–
**Analysis of workflow**									
Khan et al., 2021a, Khan et al., 2021b	Phase & step recognition	Endoscope	Endonasal	Human	50 videos	–	–	3 (7)	–
Lalys et al., 2011	Phase recognition	Endoscope	Endonasal	Human	16 videos	–	–	6 (0)	–

### 3.1 Study descriptive

The most common (53%) application of CV was the detection (Markarian et al., 2022; Philipp et al., 2022; Unadkat et al., 2022; Ramesh et al., 2021; Lee et al., 2021) or segmentation (Kalavakonda et al., 2019; Bouget et al., 2015; Deepika et al., 2023; Davids et al., 2021a) of neurosurgical tools. In 40% of those studies, additional characterization of tool characteristics were determined to calculate surrogate metrics of surgical performance. In turn, these automated performance metrics served as a tool for automatic prediction of neurosurgical skills, task success, bloodloss and dynamic brain retraction. (Markarian et al., 2022; Philipp et al., 2022; Unadkat et al., 2022; Ramesh et al., 2021; Lee et al., 2021; Kalavakonda et al., 2019; Bouget et al., 2015; Deepika et al., 2023; Davids et al., 2021b; Martin et al.). On the other hand, detection (Pangal et al., 2022; Zhou et al., 2023; Tang et al., 2022; Staartjes et al., 2021) and segmentation (Martin et al.; Witten et al., 2022) of neuroanatomical structures was the second most (35%) common application of CV. Surgical workflow analysis by phase and step classification was established in only two (12%) studies (Khan et al., 2021b; Lalys et al., 2011).

CV models were either trained on microscopic (47%) or endoscopic (30%) footage. In one study the model was trained on both microscopic and endoscopic footage. Only one study analyzed images from the neuroanatomical collection of The Neurosurgical Atlas (Witten et al., 2022). In two studies the type of images was not specified. The most frequently analyzed procedures were cranial (ca. 56%) and endonasal (ca. 33%) interventions. Only 1 study included footage from spinal surgery (Philipp et al., 2022). In one studie the type of procedure was not specified.

The median size of datasets was 19 videos (Q1 = 16, Q3 = 50). In the majority of cases, private datasets were established for model development, except for the study by Pangal et al. (2022)), Bouget et al. (2015) and Zhou et al. (2023), who published the Simulated Outcomes following Carotid Artery Laceration (SOCAL) (Pangal et al., 2021), NeuroSurgicalTools (Bouget et al., 2016) and Microsurgical Aneurysm Clipping Surgery (MACS) (Weiss Open Data Server |) dataset, respectively.

### 3.2 Algorithms and functionalities

Having collected all data, applied algorithms and performances were assessed (Table 3). In terms of applied methods, our findings show that convolutional neural networks (CNN) were most frequently (65%) applied. This is a type deep learning network frequently used in image analysis tasks, in which visual information is processed by a sequence of convolutional kernels. Several different CNN architectures were applied in the reviewed studies, including Mask Region-CNNs (R–CNN) (Deepika et al., 2022; Lee et al., 2021; Davids et al., 2021a; Tang et al., 2022), Faster R-CNNs (Philipp et al., 2022; Lee et al., 2021) or Uncertainty-based Dynamic CNNs (Philipp et al., 2022) for object detection and segmentation. In some instances, Recurrent Neural Networks (RNN) (Khan et al., 2021a; Pangal et al., 2022) were implemented in conjunction with a CNN to enhance the temporal resolution of the model through the integration of temporal relations between frames.

**Table 3:** Model architecture, development and performance metrics.

Author	Algorithm	Annotation	Allocation	Hyperparameters	Pre-training	Cross-Validation	Performance metrics
**Classification models**							
Analysis of workflow							
Khan et al., 2021a, Khan et al., 2021b	CNN RNN	Frame labelling	Training: 80% Testing: 20%	Undefined	–	–	Accuracy: 91.25% (Phase) | 75.69% (Step) Precision: 91.49% (Phase) | 82.09% (Step) Recall: 89.23% (Phase) |71.98% (Step) F1 score: 91.25% (Phase) | 75.69% (Step)
Lalys et al., 2011	SVM HMM	Undefined	Undefined	Undefined	–	10-fold cross validation	Accuracy 93% Mean ER 7.1 ± 5.3%
**Detection models**							
Analysis of anatomy and critical events							
Zhou et al., 2023	Shifted Windows Transformer Model (SWIN-T)	Frame labelling	Training: 87.5% Validation & testing: 12.5%	Learning rate: 0.0003 and 0.00003 Epoch: 30 Batchsize: 64	ImageNet	4-fold cross-validation	Accuracy 87.1% Precision 79.4% Recall 48.9% F1 score 58.9%
Pangal et al., 2022	CNN (ResNet) RNN (LSTM)	Bounding-box	Training: 86% Testing: 14%	Undefined	ImageNet	–	Accuracy: 85% Sensitivity (Recall) 100% | Specificity 66% PPV (Precision) 79% | NPV 100% RMSE 295 ml (mean error −57ml, R^2^ 74%)
Tang et al., 2022	Mask R–CNN Residual network backbone (ResNet50) FPN RPN	Undefined	Training: 80% Testing: 20%	Learning rate: 0.001 (−0.0001 at *i*^*50*^) Iterations: 1000 Weight decay: 0.0001 Momentum 0.9	COCO	–	Accuracy: 89.6 Generalised IoU: 94.4% Precision: Porcine model: 94.40% Scalp incision: 84.44% Skull incision: 89.48% Dura matter-incision: 90.46%
Staartjes et al., 2021	CNN (U-Net)	Centroid	Training: 78% Validation & testing: 22%	Learning rate: 0.001 Epoch: 500	–	–	Complete overlap: 36.1% Incomplete overlap: 19.2% Incorrect overlap: 44.7%
Analysis of surgical Instruments							
Markarian et al., 2022	One-stage object detection model (RetinaNet)	Bounding-box	Undefined	Undefined	–	–	mAP 74%
Philipp et al., 2022	Uncertainty-based Dynamic CNN	Bounding-box	Training: 38% Validation: 12% Testing: 50%	Learning rate: 0.01 Weight decay = 0.1 Epoch: 500	–	–	SIM 80.1%

Author	Algorithm	Annotation	Allocation	Hyperparameters	Pre-training	Cross-Validation	Performance metrics
Unadkat et al., 2022	AutoMl Google One-stage object detection model (RetinaNet, Yolov3)	Bounding-box	Training: 87% Validation: 7% Testing: 6%	Undefined	–	–	mAP AutoMLGoogle: 70.80% RetinaNet: 66.9% YOLOv3: 52.7% Recall: 52.63%
Ramesh et al., 2021	One-stage object detection model (Yolov5)	Bounding-box	Training: 80% Testing: 20%	Epoch: 150	COCO	–	mAP 74.4% Recall 93.6%
Lee et al., 2021	Mask R–CNN Faster R–CNN One-stage object detection model (SSD)	Bounding-box	Training: 70% Testing: 30%	Undefined	COCO	–	Accuracy: Mask R–CNN: 99.53% Faster R–CNN: 99.57% SSD classifier 98.92% Pre-trained Faster R–CNN 98.74% Precision: Mask R–CNN: 98.96% Faster R–CNN: 97.27% SSD classifier 90.96% Pre-trained Faster R–CNN 90.55% Recall: Mask R–CNN: 99.24% Faster R–CNN: 97.54% SSD classifier 93.76% Pre-trained Faster R–CNN 91.35%
**Segmentation models – Semantic**							
Analysis of anatomy and critical events							
Martin et al., 2023	CNN (U-Net)	Polygon	Training & validation: 90% Testing: 10%	Undefined	–	5-fold cross-validation	IoU: 72.64% (Stage 1) Mean Reprojection error: 4.06 Mean Scaling error: 1.01 Mean center error: Microforceps 17.71 Suction 5.42 Dissector 11.74
Witten et al., 2022	CNN (ResNet) Backbone (PSPNet)	Undefined	Undefined	Learning rate: - ResNet: 0.00001 - PSPNet: 0.0001 Epoch: 300	–	–	Accuracy 91.8% Precision 85.3% Recall 77.6% IoU 82.6% Dice coefficient 90.4% F1 score 85.3%

Author	Algorithm	Annotation	Allocation	Hyperparameters	Pre-training	Cross-Validation	Performance metrics
Analysis of surgical Instruments							
Kalavakonda et al., 2019	CNN (Vanilla U-Net, VGG16, MobileNetV2)	Polygon	Training: 87.5% Testing: 12.5%	Learning rate: 0.001 Epoch: 20	ImageNet	K fold cross-validation	IoU 74.8% Dice coefficient 76.9%
Bouget et al., 2015	SMV	Polygon	Training: 51% Testing: 49%	Undefined	–	–	Accuracy: 85.8% Detection miss-rate: 15% (10^−1^ false positives per image)
**Segmentation models – Instance**							
Analysis of surgical Instruments							
Deepika et al., 2023	Mask R–CNN	Polygon	Training: 62% Validation: 8% Testing: 30%	Undefined	COCO	–	mAP 96.7% mAP for each tool class Suction: 99.3% Bipolar Forceps: 99.8% Straight Micro Scissor: 100% IoU 50%
Davids et al., 2021a, Davids et al., 2021b	Mask R–CNN	Polygon	Undefined	Undefined	COCO	“Leave One User Out" cross validation	Accuracy: 84.21% AUC: 97.7%

CNN = Convolutional Neural Network; R–CNN = Region Convolutional Neural Network; RNN = Recurrent Neural Network; SVM = Support Vector Machine; HHM = Hidden Markov Model; FPN = Feature Pyramid Network; RPN = Region Proposal Network; *i*^*50*^ = 50th iteration; mAP = Mean Average Precision; SIM = Similarity Intersection; IoU = Intersection over Union; RMSE = Root Mean Square Error; ER = Error Rate.

CNN = Convolutional Neural Network; R–CNN = Region Convolutional Neural Network; mAP = Mean Overall Precision; AUC = Area Under ROC Curve, IoU = Intersection over Union.

SMV = Support Vector Machine; R–CNN = Region Convolutional Neural Network; mAP = Mean Overall Precision; AUC = Area Under ROC Curve, IoU = Intersection over Union.

Whereas these are all examples of two-stage object detectors of CNN, one-stage detectors included YOLO (Unadkat et al., 2022; Ramesh et al., 2021) and SSD (Lee et al., 2021). As the name implies, one-stage detection models perform object classification and localization in a single stage, essentially through the mechanism of foreground-background discrimination (Zhang and Cloutier, 2022; Liu et al., 2016). Two-stage detection models on the other hand will boost their overall accuracy by first performing a region proposal procedure, where the image is scanned for regions of interest (ROI) that harbor potential relevant objects. In a second stage, object classification and localization is performed on the pre-defined ROI's (Carranza-García et al., 2021).

Other methods that were employed for image analysis, other than CNNs, included Support-Vector-Machines (SVM) (Bouget et al., 2015; Lalys et al., 2011) and Hidden Markov Models (HMM) (Lalys et al., 2011), which are typically used for classifying tasks. The latter method is particularly relevant in cases where the temporal considerations hold significance (e.g. phase recognition). In contrast to the former studies, the more recent study of Zhou et al. (2023) utilized a Transformer model that, despite its conventional application in natural language processing (NLP), has recently gained interest in the field of CV (Liu et al., 2021; Dosovitskiy et al., 2020).

Among the different studies, there was a wide variation in data allocation for training (50–90%), validation (7–12.5%) and testing (10–50%). Just under half of the models were pre-trained with (non-surgical) public datasets such as COCO and ImageNet. Only 6 studies included a cross-validation step in their model development. Model hyperparameters used for model training, were rarely mentioned and were therefore not further assessed.

### 3.3 Model performances

**Fig. 4:**
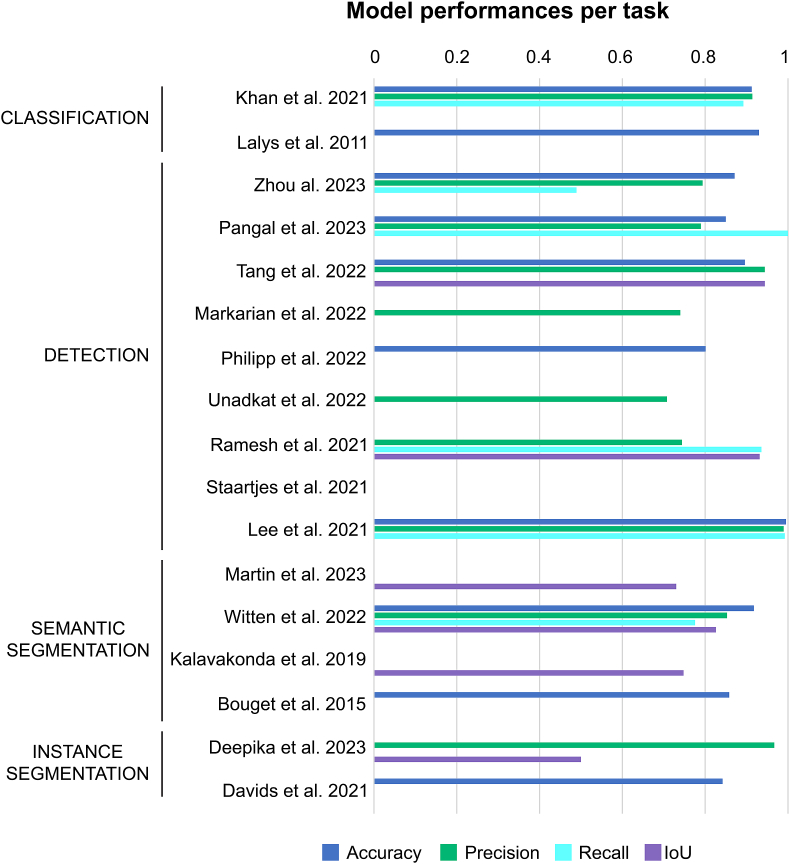
Model performances per task.

Following comparison of the model frameworks, study results were examined in more detail (Fig. 4). With regards to model performances, there was an important heterogeneity in the way authors reported outcomes. Accuracy and precision was disclosed in resp. 9 and 10 out of 17 studies, whereas Recall and IoU only in resp. 5 and 7 out of 17 studies. The remaining performance metrics (Dice coefficient, F1-score, AUC, etc.) were significantly underrepresented.

#### 3.3.1 Classification models

In terms of phase & step classification accuracy, Lalys et al. (2010) (93%) demonstrated the highest performance, compared to Khan et al. (2021a) (phase: 91.25%, step: 75.69%). On the other hand, Khan et al. (2021a) demonstrated a precision ranging from 91.49% to 82.09% with the recall ranging from 89.23% to 71.98% with respect to phase and step detection. Lalys et al. (2010) did not provide any precision nor recall scores.

#### 3.3.2 Detection models

In terms of accuracy, Tang et al. (2022) (89.60%) demonstrated slightly higher performances, compared to Zhou et al. (2023) (87.1%) and Pangal et al. (2022) (85.00%) for the detection of anatomical structures and critical events. On the other hand, the reported accuracies of Lee et al. (2021) (98.74–99.57%) for instrument detection surpasses the former results.

With regard to anatomical structure and critical event detection, Zhou et al. (2023) demonstrated a precision of 79.40% at a recall of 48.9% and Pangal et al. (2022) reported a precision of 79.00% at a recall of 100%. On the other hand, Tang et al. (2022) demonstrated a precision of 89.7%, however their recall was not specified.

With regard to instrument detection, Lee et al. (2021) displayed high precision of 90.55–98.96% at a recall of 91.35–99.24%. Ramesh et al. (2021) reported a precision of 74.40% at a recall of 93.60% and Unadkat et al. (2022) 70.80% at a recall of 52.63. Markarian et al. (2022) demonstrated a precision of 74.00%), however their recall was not specified.

#### 3.3.3 Segmentation models

In terms of degree of correspondence between the predicted regions of anatomical structures and ground truth, Witten et al. (2022) (82.60%) displayed a higher IoU compared to Martin et al. (Martin et al.) (73.00%). With regard to instrument segmentation, Kalavakonda et al. (2019) (74.80%) displayed a higher IoU compared to Deepika et al. (2022) (50.00%).

The work of Staartjes et al. (2021) was not included in the class-wise performance comparison since standardized performance metrics were not applied to describe their model output. Their model displayed a complete overlap between the predicted anatomical structure and ground truth in 36.1%, whereas the remaining predictions were either partially overlapping (19.2%) or incorrect (54.6%).

Clustered bar plots displaying the performance of the different studies with respect to accuracy (blue), precision(green), recall (turquoise) and IoU (purple). Studies are divided into classification, detection and segmentation models.

### 3.4 Automatic skill assessment

Two studies utilized surrogate performance metrics, derived from CV-based recognition of instruments in surgical images/videos, for automatic assessment and characterization of surgical skills. A first study examined suturing segments of surgical videos to assess surgical skillfulness by means of tool handling metrics and microscope handling metrics (Deepika et al., 2023). They reported a significant lower velocity, acceleration and jerk of surgical tools amongst experienced surgeons as opposed by novice surgeons. Moreover, more experienced surgeons displayed a higher fluency and efficiency of movements expressed by a reduction in pathlength, inter tool-tip distance and increased bimanual tool usage. In terms of time usage, analysis revealed a significant reduction in the idle time amongst experienced surgeons. Additional skill characterization was established through examination of the microscope application, revealing a reduction of microscope adjustments with the surgeon's experience. These findings are in line with results from an earlier study which assessed the performance of surgeons with varying experience on arachnoid dissection in a brain phantom model (Davids et al., 2021a, Davids et al., 2021b). Here, increasing surgical experience was associated with a reduction of the average velocity, jerks, inter tool-tip distance and the total time of tool absence yet an increase in bimanual tool handling. Their model was capable of differentiating expert from novice surgeons with an accuracy of 84.2% and an AUC of 0.977.

Apart from automatic assessment and characterization of surgical skills, the correlation of the abovementioned surrogate performance measures and surgical outcomes remains to be investigated.

## 4 Discussion

### 4.1 New era of surgical intelligence

To our knowledge this is the first systematic review that summarizes and compares all studies using CV for automatic analysis of neurosurgical videos/images. Despite the fact that CV is already well established in other surgical domains, the field is currently only in its early development in neurosurgery. Therefore, it is interesting to consider the evolution of the performances as well as the evolution of the applications and functionalities of the models.

While most studies report on basic scene understanding, in terms of tool or phase detection, others were aimed at more advanced analysis. This includes automated surgical skill assessment, calculated by means of instrument handling metrics such as position, usage time, motion trajectory etc. Ramesh et al. (2021). and Deepika et al.^57 34^demonstrated how these metrics help to provide insight into the intricacies of surgical manipulations. Furthermore, Davids et al. (2021a) managed to automatically classify the level of microsurgical skills purely on the basis of automatic analysis of a surgeon's instruments.

More recently, Martin et al. (Martin et al.) integrated instrument and anatomical features to tackle the aspect of brain tissue deformation resulting from surgical instruments. As such they could provide operators with artificial haptic feedback on their tool employment. On the other hand, studies by Pangal et al. (2021a) and Kugener et al. (2022) focused more on task-effectiveness and outcome prediction in complication management. They established a deep learning model (SOCALNet) able to predict the amount of blood loss and task success in the event of a carotid artery laceration during endonasal endoscopic surgery (Pangal et al., 2022).

Our findings show that CV models can identify and assess discrepancies in neurosurgical performance with high accuracy (84.2%). Above all, the performance grading is established in an objective manner, contrary to existing assessment models (e.g. OSATS, GRS, etc.) that are prone to subjectivity due to involvement of a human evaluator. Moreover, evaluation may cover a wider range of performance criteria, thus providing a more adequate representation of the operator's performance. In terms of time investment, automatic assessment may facilitate the work of the examiner, allowing for more time to be spent on education itself rather than assessment.

As such, automatic assessment of the quality of a surgical performance paves the way for proficiency-based surgical progression at hand of precise directives, tailored to an operator, with respect to a specific benchmark to ensure deliberate surgical training. Moreover, the recognition of premonitory signs of complications or acquiring haptic feedback could enhance surgical practice, reducing errors by adjustments of the surgeons actions.

### 4.2 Performance

Although all studies reported quantitatively on the obtained result, a true comparison between the different studies was challenging due to inter-study variation of used data and reported performance metrics. This observation stresses the importance of a standardized application of metrics to evaluate CV models.

Classification models were only applied by studies analyzing surgical workflow, namely phase and/or step recognition. This is related to the fact that phase and step analysis focusses on time efficiency and procedural order rather than detailed surgical features such as tool handling. Given that no object localization is required, detection or segmentation models are not necessary.

Lalys et al. (2011) showed slightly better results in terms of phase detection accuracy in comparison to Khan et al. (2021a). However, they did not mention any precision nor recall. Hence true superiority could not be assessed. Regardless of the performance results, the framework of Khan et al. (2021a) demonstrates a higher functional resolution in the sense that they provided the possibility for identification of the surgical step in addition to the phase classification. They achieved this by implementing a CCN architecture in conjunction with an RNN.

Contrary to classification models, detection models were principally used for characterization and localization of surgical tools. Since multiple types of tools are applied in surgery, accuracy is of great importance given that each tool has a different function and connotation (e.g. bipolar is often used to stop surgical bleeding). The second most application was the detection of surgical hemorrhages.

Overall, Lee et al. (2021) demonstrated the highest overall performance, which stand in contrast to the results reported by Philipp et al. (2021), Ramesh (Ramesh et al., 2021) et al. and Unadkat et al. (2022) A notable difference with other studies was that the size of their dataset was considerably larger (950 videos), which may have contributed to the higher accuracy, precision and recall. Interestingly, they performed a case comparison between different model architectures, of which the Mask R–CNN yielded the best overall results.

Studies analyzing critical events (Pangal et al. (2022) and Tang et al. (2022)) and anatomical structures (Zhou et al., 2023)) displayed superior precision as opposed to Philipp et al. (2021), Ramesh (Ramesh et al., 2021) et al. and Unadkat et al. (2022), which focused on surgical tool detection and localization. In this framework, precision is of utmost importance given that the localization of a hemorrhage or an aneurysm will determine how or where a surgeon must act upon. The reported accuracies amongst the studies analyzing adverse events and anatomical structures are fairly similar. Although inferior accuracies are reported compared to Lee et al. (2021), one must take into account that anatomical structures and especially adverse events show a vast variation from one patient to another. Surgical tools on the other hand display similar sizes, shapes, colors and textures which makes them an easier target for automatic recognition. Thus, lower outcomes must be nuanced. In terms of model preferences, CNN's were applied in the majority of the above mentioned studies.

Similar to detection models, segmentation models were used primarily for characterization and localization of surgical tools. However, the function of the segmentation models differ in the sense that information about the pose of instruments can be provided in addition to its location. As mentioned earlier, instance segmentation differentiates multiple instances whilst semantic segmentation only differentiates objects of interest from the background. In this regard surgical skill assessment was solely analyzed in the instance segmentation models, which allows for tool/hand specific metrics. Once more, R-CNNs were the algorithm of preference.

While no model can be classified as the absolute best, there are noteworthy observations regarding the utilization of models and their respective results. For instance, we noticed a predominant use (65%) of CNN architectures across all 3 task modalities. In tool detection and segmentation, CNNs displayed highest performances. Especially Mask R-CNNs, which were the most frequently employed CNN framework. Similarly, in the models that focused on anatomical structures, Mask R-CNNs was at the higher end of the reported performances. Studies reported the use of largely varying auxiliary strategies to improve performance, including pre-training, data-cross validation, image pre/post-processing, and so forth (Ikeuchi and Ikeuchi, 2014).

### 4.3 Machine vs. Human

Whilst analyses often revolve around the absolute values of the outcome result of a model, we often fail to provide insight in the relative performance of the CV algorithms against that of human operators. A common misconception is that de values we strive to obtain, 100% accuracy/precision/recall/ …, are the representations of the performance of the human visual system. This is incorrect in the sense that these absolutes merely are in accordance to the ground truth, i.e. labels that are given by one or more human experts in a controlled setting. Thus, they are not equivalent to the average performance of neurosurgeons. To truly capture the benefit of an automated system, comparison must be made between human and machine, both in terms of accuracy and time investment.

For instance, Pangal et al. (2022) assessed the task success and blood loss prediction of their model against 4 neurosurgeons. Results demonstrated that expert surgeons were less successful (sensitivity 82%, specificity 55%, PPV 69% and NPV 71%) in predicting the outcome of surgical hemorrhage from 1 min of video as opposed to the CV model (sensitivity 100%, specificity 66%, PPV 79% and NPV 100%). The most notable observation was that expert surgeons displayed poorer performance at the intermediate skill levels as opposed to the SOCALNet model, while their performances at the low and high skill levels was highest. As such, Pangal et al. draw notice to the notion that CV models may possess superior aptitude for the assessment of surgical videos that involve more ambiguous levels of skillfulness, thus allowing for a more consistent assessment across a varying degrees of skillfulness.

So how do these findings translate to the clinical practice? As was discussed earlier, physicians are increasingly experiencing difficulties in analyzing the vast amount of data presented upon the treatment of a patient. An issue which is amplified by the never-ending shortage of time. Our hunger for knowledge has surpassed our own capacities, leaving behind a vast amount of data untapped. In this respect, CV has shown to offer possibilities to assimilate the information that resides within this data. As such CV models are implemented for diagnostic purposes, pre-operative planning, outcome prediction (Panesar et al., 2020; Senders et al., 2018a, 2018b; Danilov et al., 2020a, 2020b). Given the proficient data analysis capabilities of CV, the utilization of automatic analysis may facilitate context awareness of surgeons through live feedback on surgical workflow, recognition of hazardous areas, impending complications. Essentially, the benefit of CV is substantiated by the consistency, objectiveness, tirelessness of the system and most importantly by the ability to assimilate multiple processes/activities/objects simultaneously during analysis.

In this regard, the surgeon might seem obsolete. However, in the prospect of creating computer-vision systems for the automatic analysis of surgical data, it is important to underline that the implementation of the technology ought to be regarded as an adjuvant or an extension of the human operator rather than a replacement. Whilst the technology may display higher accuracies or precision, humans will inherently show higher understanding of the surgical circumstances or the clinical relevance of the observation, which is indispensable for interpreting the predictions generated by these models. The most evident example of this is the lack of quality control mechanisms within computing technologies that can verify the logical consistency of their outputs. In light of these elements we should strive collaborative relationship between humans and machines, rather than a man-versus-machine paradigm. This is due to the reason that human control remains critical as apparently flawless software remains susceptible to producing errors at some point in time.

### 4.4 Pitfalls

#### 4.4.1 Data annotation

As the expression “garbage in, garbage out” elegantly states, the use of high-quality data is paramount in the development of CV models. Although we produce significant amounts of data, the vast majority (ca. 90%) of it is unstructured (medical notes, medical images, etc.) and require some form of encoding or characterization prior to any examination (Raju et al., 2020). Moreover, for some data types such as videos/images, additional labeling is required in order for them to be analyzed (Senders et al., 2018c), which often is the bottleneck for model development as result of lack of time and/or qualified personnel (Markarian et al., 2022; Bydon et al., 2020).

Objects can be annotated through the process of placing bounding-boxes as closely as possible around a target object instances, thereby providing localization and ground truth label. An alternative approach is segmentation, where objects are delineated by manually tracing the outline of the structure (Pangal et al., 2021b; Ward et al., 2021d). Although bounding boxes are easy to use and therefore often applied, literature suggests that segmentation could contribute to better detection performances (Pangal et al., 2021b; Mullen et al., 2019). For phase classification, annotation is performed through frame-wise labeling. Strategies that may facilitate the process of annotation and improve the fidelity of annotations are: down-sampling the annotated data, outsourcing the annotation process to specialized firms who possess dedicated tools, use of software that enable interpolation and copy-pasting of annotations and the use of an annotation protocol (Ward et al., 2021a, 2021d; Pangal et al., 2021b).

#### 4.4.2 Dataset

Apart from the quality of the data, the model robustness also relies on the quantity and variability of the data. The larger and the more heterogenous the dataset, the better the model performs when presented with new data (Panesar et al., 2020; Senders et al., 2018a; Bamba et al., 2021b). Generally, the rule of thumb is to obtain around 1000 images per class (Ikeuchi and Ikeuchi, 2014). For model training, videos/images of real operations are preferably used instead of phantom or cadaver experiments, as they are less likely to mimic all the properties of real operating conditions. The performance of the model can be additionally improved using data augmentation techniques, which allow to artificially increase the size of train data set by introducing geometrical or intensity distortions, such as the adjustment of brightness or image contrast, flipping, random rotations or affine geometrical transformations. Data augmentation allows to improve the accuracy and robustness of the model by enhancing the ability of your model to recognize new variants of the training data (Shorten and Khoshgoftaar, 2019).

To set up an experimental design of a CV model, data is conventionally split into a training, testing and ideally a validation set. As per definition, the training set allows the model to learn from the data. A validation set allows a model to optimally adapt its hyperparameters with purpose of increasing its prediction performance. Conventionally, testing data consists of entirely novel data, not yet seen during training, with purpose of performing an unbiased evaluation of the final model performance (Tarang, 2017). In terms of data allocation a 2-1 distribution is recommended for training and testing, respectfully (Senders et al., 2018a), albeit in scenarios of limited data a 6-1 distribution has also been suggested (Ross et al., 2018).

#### 4.4.3 Model

There are multiple factors that may cause interference in the functioning of ML models such as smoke, image blurring, reflection and so on. Implementation of CNNs, appears to cope better with such image distortions than other algorithms. On this account, CNNs have displayed the best result in the literature so far when it comes down to object detection. This finding is also reflected in our results. However, a problem CV currently struggles to account for is the lack of temporal context. Often, the integration of a RNN (Khan et al., 2021a; Lee et al., 2021; Deepika et al., 2023; Davids et al., 2021a; Tang et al., 2022) or a long-short term memory (LSTM) (Pangal et al., 2022) network helps to perceive this temporal context, which improves model performances.

### 4.5 Limitations

First, since our search syntax consisted of English terms, this systematic review may include a potential bias towards English language publications. As a result, it is possible that relevant studies published in languages other than English, were not included in the review. However, the majority of studies in the field of image computing are published in English, thus the potential impact of this limitation on the overall findings is likely to be minimal.

A second limitation of our study was that we were unable to provide a one-on-one comparison between models as consequence of differences in datasets and discordant use of performance metrics. Despite this limitation, a descriptive analysis was conducted to elucidate the mechanisms, benefits and functionalities of various CV models with respect to their function.

Finally, it is important to note that many studies continue to rely on their own development datasets, which are often highly selective (ideal lighting, no obscuration of objects, perfect camera settings, clean anatomy, etc.) in nature. As a result, the performance results reported are likely to be disproportionately optimistic. To prevent this, studies should ideally have to be tested on a fully independent dataset.

### 4.6 Recommendations for the future

It is important to underline that the studies we have reviewed were primarily conducted within a preclinical environment, frequently focused on a single type of surgical procedure (e.g. suturing). Consequently, future investigations should evaluate the application of these CV models in the clinical setting to ascertain their applicability, efficacy and reliability. In this context, we strongly advocate for the use of open-source, as it is indispensable for improvement in software development and implementation (Senders et al., 2018c).

As data acquisition and lack of annotated data are often a bottleneck in the development of qualitative CV models, initiatives should be undertaken to develop open databases (Rodrigues et al., 2022). Additionally, standardization of taxonomy of adverse events, surgical tasks and operations should be implemented to ensure cross-site applicability of diverse sources of neurosurgical data and facilitate comparability of model outputs. Such measures will enable benchmarking of model performances and ultimately leading to the external validation of models. In this regard, we emphasize the crucial importance of a unified annotation framework to guarantee consistent data quality (Pangal et al., 2021b; Meireles et al., 2021).

This, in turn, raises the issue of standardization of outcome results, which is paramount for the validation, comparison and implementation of CV models. Prior to developing these models, it is crucial to address the specific purpose they serve (classification, detection, segmentation), as this dictates the appropriate performance metrics to be employed. However, metrics are often interchanged (e.g. precision and accuracy) even though they refer to different model characteristics. This raises concerns regarding the interpretability of the reported outcomes.

Furthermore, it is crucial to observe that the meaningful comparison of metrics takes place exclusively when carried out at equivalent levels of their corresponding metric (for instance, precision and recall). Similar to sensitivity and specificity, adjustments to the threshold of one value will cause the other to change in an inverse relationship. Hence, for instance, presenting precisions in isolation or comparing performances in absence of identical recall scores lacks significance. To facilitate straightforward intercomparisons, the use of metrics such as Area Under the operator Curve (AUC) could prove to be of value since they offer the possibility to distill outcomes into singular numerical values.

In response to this challenge, a multi-stage Delphi process on metrics was conducted by an international consortium, which issued a series of recommendations regarding the use of metrics for reporting results of CV models (Maier-Hein et al., 2022). A summary of their proposed metrics can be found in Table 1.

All these factors collectively contribute to the advancement of the performance of CV and its integration in our practice, a progress that can further be enhanced through incorporation of these elements in open challenges focusing on neurosurgical image analysis, in which various algorithms proposed by the participating contenders are evaluated on the same dataset and using consistent performance evaluation. This will stimulate the exchange of expertise and ultimately promote technological innovation within the field of neurosurgery.

## 5 Conclusions

To our knowledge, this is the first systematic review providing a comprehensive summary of the state-of-the-art methodologies in CV for neurosurgical instruments, anatomy and workflow analysis. Findings from our qualitative analysis provided a groundwork for a number of recommendations in regard to model development.

In spite of the high technical and anatomical complexity of neurosurgical scene interpretation as opposed to robotic or laparoscopic interventions, our result demonstrate that CV models can effectively detect and differentiate tools, phases and neuroanatomical structures with accuracies above 95%. Furthermore, automatic recognition of tools can contribute to the characterization and objective assessment of surgical performance, which opens numerous prospects for neurosurgical training. CV models have also proven to be a valuable asset in increasing intra-operative safety through the detection of blood loss and quantification of brain retraction induced by instrument interaction.

## Funding

This research did not receive any specific grant from funding agencies in the public, commercial, or not-for-profit sectors.

## Authorship contribution

FB, JV, JC, FV, MB: Conception and design of the study. FB, JV, JC, FV: Acquisition of data. FB, JV, JC, MB: Analysis and interpretation of data. FB, JV, JC, FV, JD, JFC, MB: Drafting the manuscript. FB, JV, JC, FV, JD, JFC, MB: Critical revision of the manuscript. All listed authors have made substantial contributions to the presented study, read, and approved the final submitted manuscript.

## Declaration of competing interest

The authors report no conflict of interest.

## Appendix

The exact search query that was applied in Pubmed was: ("Surgical scene"[Title/Abstract] OR "Tool"[Title/Abstract] OR "Instrument"[Title/Abstract] OR "Workflow"[Title/Abstract] OR "Phase"[Title/Abstract] OR "Action"[Title/Abstract] OR "Skill"[Title/Abstract] OR "Performance"[Title/Abstract]) AND ("Neurosurgery"[All Fields] OR "Neurosurgical"[All Fields] OR "Neurosurgeon"[All Fields]) AND ("Surgery"[All Fields] OR "Procedure"[All Fields] OR "Intervention"[All Fields] OR "Operation"[All Fields]) AND ("Computer vision"[All Fields] OR "Machine vision"[All Fields] OR "Vision-based"[All Fields] OR "Machine learning"[All Fields] OR "Deep learning"[All Fields] OR "Image analysis"[Title/Abstract] OR "Video analysis"[Title/Abstract])
